# Emotional meanings reported by patients about their life experiences under the following in the Watch and Wait protocol: A qualitative study in a Brazilian surgery university specialized outpatient service

**DOI:** 10.1192/j.eurpsy.2024.1347

**Published:** 2024-08-27

**Authors:** M. R. Suedt, S. A. Silva, A. C. O. Bispo, R. S. E. Sant’Ana, L. M. Guerra, C. A. R. Martinez, J. B. C. Carvalheira, C. S. P. Lima, E. R. Turato

**Affiliations:** ^1^ Lab of Clinical-Qualitative Research; ^2^Departamento de Cirurgia, State University of Campinas, Campinas, Brazil

## Abstract

**Introduction:**

Health Psychology is aggregated to clinical studies providing physicians, nurses, and psychotherapists with psychodynamics of sick persons, facilitating interpersonal relationships and greater adherence to treatments. How do people deal with illness and treatment from what they symbolize in experiences of becoming ill? Watch & Wait Protocol for patients with rectal cancer is an active surveillance as an alternative approach in surgical medical management. Patients are followed with physical examinations, endoscopy, and imaging. Observation carried out through periodic examinations aims to avoid surgery stage while rectal cancer is maintained.

**Objectives:**

To interpret emotional meanings attributed by patients, after adhering to the W&W protocol for rectal cancer, to life experiences of watching and waiting for the disease course.

**Methods:**

Clinical-Qualitative Method (Turato. Portuguese Psychos. J, 2000 2(1): 93-108). For data collection, the first author used Semi-Directed Interview with Open-ended Questions In-Depth and Field Notes, after acculturation. Sample closed by information saturation (Fontanella et al. Cad Saude Publica. 2008; 24(1): 17-27). Interviews conducted by the first author, a female psychologist. We employed the Seven Steps of the Clinical-Qualitative Content Analysis (Faria-Schützer et al. Cien Saude Colet. 2021; 26(1): 265-274) to construct categories. Theoretical framework was the Balintian Medical Psychology. Findings were validated by peer reviewers from Lab of Clinical-Qualitative Research.

**Results:**

Sample had 10 patients, 3 female and 7 male, from 52 to 77 years. Interviews carried out from October 2022 to March 2023. We constructed 03 categories: 1) Fate out of hands - “I get sick just coming in here.” There is an apprehension experienced in each medical evaluation to check the clinical condition because the symbol of waiting is not having the own destiny in the hands. 2) Psychic defence - “Sometimes I even thought if I had to live on a grant for the rest of my life or die!” Imagining the worst is a psychic defence because if this probability occurs, the mind has already begun its elaboration. 3) Life upside down - “I was going to have the surgery, use a bag, my life was going to be upside down.” Anxiety generated by waiting is a mental disorganizing.

**Conclusions:**

Attitudes of observing and waiting carry different symbolisms to those who work with scientific thinking and who experience the observation of their own disease and the wait for what conduct they will receive. Observing oneself in illness requires acceleration of changes in ego identity. Waiting in front of illness asks the ego to think the worst. It is not a volitional choice. Preparing for the worst is a defensive necessity in the emotional sphere to avoid surprises that take to mental rupture.

**Disclosure of Interest:**

None Declared

